# Supplementary figures and images for: Nociceptive withdrawal reflexes of the trunk muscles in chronic low back pain
Source: PLoS One. 2023 Jun 14;18(6):e0286786. doi: 10.1371/journal.pone.0286786 (PMC10266613; doi:10.1371/journal.pone.0286786)

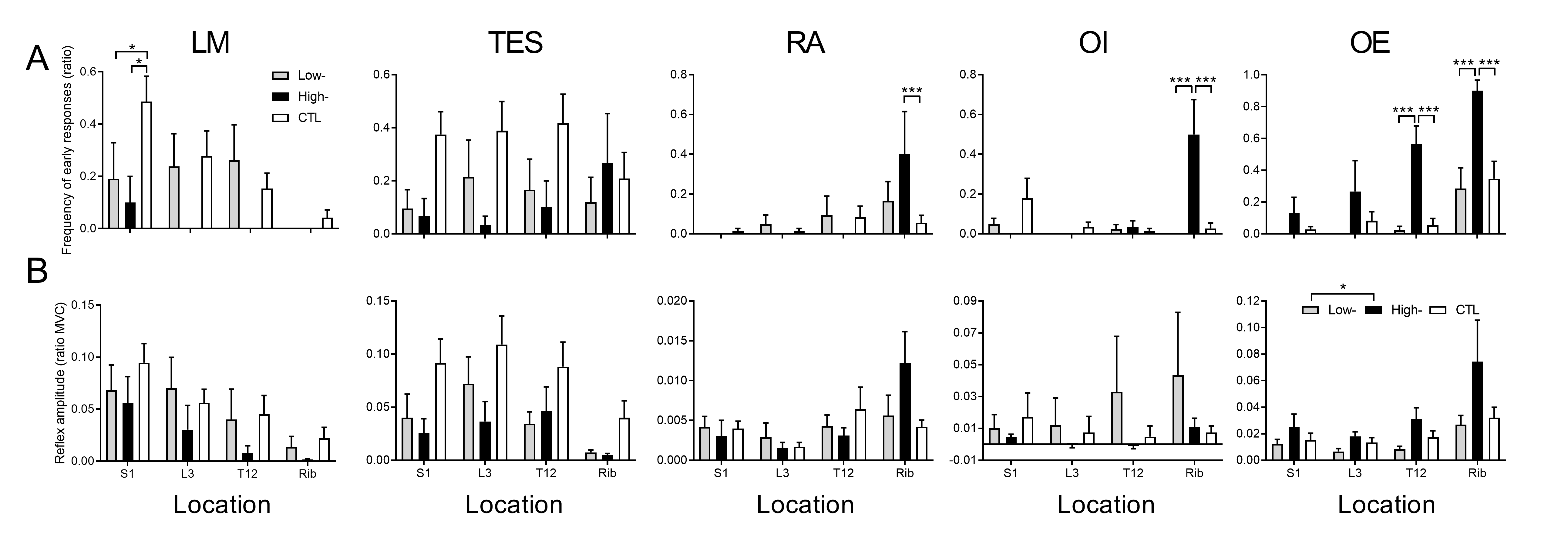

Supplement: S1 Fig — (A) Frequency of occurrence of early responses(ratio), and (B) amplitude of late responses for LM, TES, RA, OI and OE. LM: Lumbar multifidus; TES: Thoracic erector spinae; RA: Rectus abdominus; OI: Obliquus internus abdominis; OE: Obliquus externus abdominis; S1/L3/T12: Spinous processes of S1, L3 and T12. *p<0.05; **p<0.01; ***p<0.001. (TIF) [file pone.0286786.s001.tif]

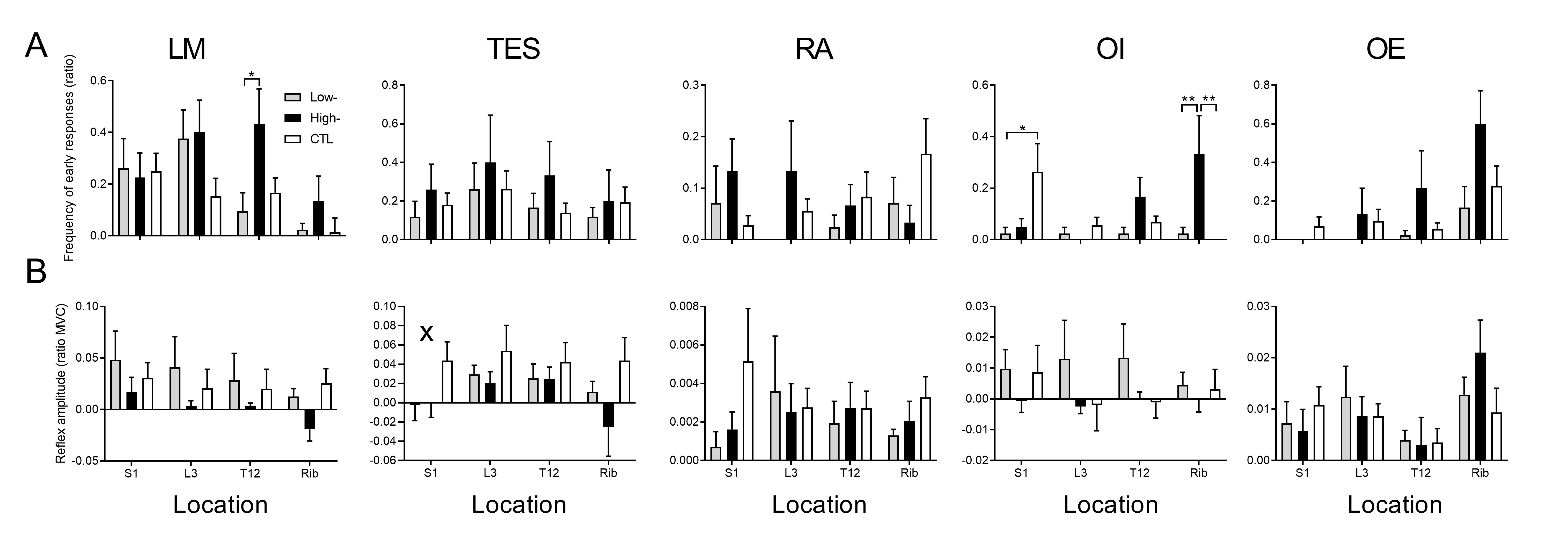

Supplement: S2 Fig — (A) Frequency of occurrence of early responses (ratio), and (B) amplitude of late responses for LM, TES, RA, OI and OE. LM: Lumbar multifidus; TES: Thoracic erector spinae; RA: Rectus abdominus; OI: Obliquus internus abdominis; OE: Obliquus externus abdominis; S1/L3/T12: Spinous processes of S1, L3 and T12. *p<0.05; **p<0.01; ***p<0.001; X—Subroup x Site interaction was observed but without Subgroup differences detected by pairwise comparisons, and comparisons between locations (within-group) are reported in Table A in S1 Text. (TIF) [file pone.0286786.s002.tif]
