# Supplementary material for: Nociceptive withdrawal reflexes of the trunk muscles in chronic low back pain
Source: PLoS One. 2023 Jun 14;18(6):e0286786. doi: 10.1371/journal.pone.0286786 (PMC10266613; doi:10.1371/journal.pone.0286786)
Supplement: S1 Text — (DOCX) [file pone.0286786.s003.docx]

**Supporting information**

**Results of subgroup analyses**

*Between group comparison of motor strategies and receptive field induced by noxious stimulation*

For presentation of the results, between-group pairwise comparisons for significant Subgroup × Site interactions are reported in S1 and S2 Figs., and GEE statistics and between-site pairwise comparisons are reported in Supplementary Table 1.

*Motor strategies - lying*

Differences in *motor strategies* were considered by evaluating the muscle response to each stimulation site. Subgroup × Site interaction was significant for the frequency of occurrence of the early response of LM (Wald χ^2^ (6) =14.7; p=0.02), RA (Wald χ^2^ (6)=15.8; p=0.02), OI (Wald χ^2^ (6)= 35.5; p=0.001) and OE (Wald χ^2^ (6) = 16.3; p=0.01).

All between-group differences (p-values) are presented in S1 Fig.. Post hoc analysis revealed several differences in *motor strategy* between groups. For stimulation of the Rib, early responses of abdominal muscles (RA, OE and OI) were more frequent (S1B Fig.) for High- than Low-threshold CLBP and CTL. LM responses were smaller in the Low-threshold than Control group. In response to T12 stimulation, OE early responses were more frequent (S1B Fig.) for High- than Low-threshold and CTL. S1 stimulation was characterized by less frequent LM in Low- and High-threshold than control.

A main effect of Subgroup represents an effect across all sites. OE late response amplitude (Main effect: Subgroup - Wald χ^2^(2)=7.4; p=0.03 - Fig. 2C) was larger.

*Receptive field - lying*

*Receptive fields* were considered by comparison of frequency/amplitude of responses of each muscle between stimulation sites. Within-group post-hoc comparisons are detailed in S1 Table. Early responses of LM presented different patterns between CLBP subgroups and the CTL group suggesting different muscle *receptive fields* (see S1A Fig.; S1 Table). For example, LM early responses were more frequent in CTL participants after S1 stimulation (i.e., stimulus that LM can most effectively withdraw the body away from) than any other site, whereas no differences between stimulation sites were observed for either CLBP subgroup (S1A Fig.; S1 Table).

| S1 Table: Between-location, within-subgroup pairwise comparisons for significant Subgroup × Location interaction | | | | | | | | | | | | |
| --- | --- | --- | --- | --- | --- | --- | --- | --- | --- | --- | --- | --- |
|  | Position | Muscle | Wald χ^2^; p | Site | | Low- | | High- | | | CTL | |
| Late reflex amplitude | Sitting | TES | 13.1; 0.04 | Rib | L3 | - |  | 0.01 | ↓ | - | |  |
|  |  |  |  |  | T12 | - |  | 0.001 | ↓ | - | |  |
| Occurrence of Early response | Lying | LM | 14.7; 0.02 | S1 | L3 | - |  | - |  | 0.04 | | ↑ |
|  |  |  |  |  | T12 | - |  | - |  | <0.001 | | ↑ |
|  |  |  |  |  | Rib | - |  | - |  | <0.001 | | ↑ |
|  |  |  |  | L3 | Rib | - |  | - |  | 0.01 | | ↑ |
|  |  | RA | 15.8; 0.02 | Rib | S1 | - |  | <0.001 | ↑ | - | |  |
|  |  |  |  |  | L3 | - |  | <0.001 | ↑ | - | |  |
|  |  |  |  |  | T12 | - |  | <0.001 | ↑ | - | |  |
|  |  | OI | 35.5; 0.001 | Rib | S1 | - |  | <0.001 | ↑ | - | |  |
|  |  |  |  |  | L3 | - |  | <0.001 | ↑ | - | |  |
|  |  |  |  |  | T12 | - |  | <0.001 | ↑ | - | |  |
|  |  | OE | 16.3; 0.01 | Rib | S1 | 0.04 | ↑ | <0.001 | ↑ | <0.001 | | ↑ |
|  |  |  |  |  | L3 | 0.04 | ↑ | <0.001 | ↑ | 0.001 | | ↑ |
|  |  |  |  |  | T12 |  |  | 0.04 | ↑ | 0.005 | | ↑ |
|  | Sitting | LM | 13.1; 0.04 | Rib | S1 | - |  | - |  | 0.01 | | ↓ |
|  |  |  |  |  | L3 | 0.001 | ↓ | - |  | - | |  |
|  |  |  |  |  | T12 | - |  | 0.049 | ↓ | - | |  |
|  |  |  |  | L3 | T12 | 0.02 | ↑ | - |  | - | |  |
|  |  | OI | 21.7; 0.001 | S1 | L3 | - |  | - |  | 0.02 | | ↑ |
|  |  |  |  |  | T12 | - |  | - |  | 0.03 | | ↑ |
|  |  |  |  |  | Rib | - |  | - |  | 0.001 | | ↑ |
|  |  |  |  | L3 | Rib | - |  | 0.02 | ↓ | - | |  |
| Low/High-: Low/High-threshold low back pain subgroup; CTL: Control group; LM: Lumbar multifidus; OE/OI: Obliquus externus/internus; RA: Rectus Abdominus; S1; Sacrum; L3/T12; spinous process of L3/T12; ↑: the site in the left column is larger/earlier than the site in the right column; ↓: the site in the left column is smaller/later than the site in the right column. | | | | | | | | | | | | |

*Motor strategies - Sitting*

There was a significant Subgroup × Site interaction for the frequency of occurrence of early responses of LM (Wald χ^2^ (6)=13.1; p=0.04) and OI (Wald χ^2^ (6)= 21.7; p=0.001).

Between-group differences (p-values) are presented in S2A Fig.. Noxious stimulation over the Rib evoked a more frequent OI early response in sitting in High- than Low-threshold CLBP and CTL (S2A Fig.). Stimulation of S1 evoked more frequent OI early response in CTL than Low-threshold subgroup (S2A Fig.). In response to T12 stimulation, the early LM response was more frequent in High- than Low-threshold (S2A Fig.).

*Receptive fields - Sitting*

The organization of receptive field in sitting differed between subgroups (see Suppl. Table 1 for details within-group comparisons). For instance, High-threshold CLBP presented more frequent early LM responses after T12 than Rib stimulation, Low-threshold CLBP had more frequent LM activation after stimulation of L3 than T12 and Rib, and CTL presented more frequent occurrence of early LM response after S1 stimulation than Rib (S2A Fig.; S1 Table 1). This suggests that each subgroup presents with a different LM receptive field in response to noxious stimulation. In addition, S1 stimulation produced more frequent OI occurrences than the stimulation of any other site in CTL whereas there is no difference between sites for High and Low-threshold subgroups (S1 Table).
