# Supplementary material for: Nociceptive withdrawal reflexes of the trunk muscles in chronic low back pain
Source: PLoS One. 2023 Jun 14;18(6):e0286786. doi: 10.1371/journal.pone.0286786 (PMC10266613; doi:10.1371/journal.pone.0286786)
Supplement: Attachment — Submitted filename: Response to reviewers.docx [file pone.0286786.s005.docx]

PONE-D-22-26918R1

Nociceptive withdrawal reflexes of the trunk muscles in chronic low back pain

**Response to Reviewer #1:**

I thank the authors for critically revising the manuscript. I think the manuscript has significantly improved with this last revision. My main concerns regarding reflex thresholding and elicitation have been further elaborated. I, however, still have a few comments which should be addressed.

RESPONSE: We are pleased that Reviewer 1 appreciate the effort done to revise the manuscript.

Q: Response 2: regarding NWR threshold determination, while it may be time consuming to map the NWR threshold for all stimulation sites, I do not believe that the thresholding is as painful as the reflex recording itself, which uses 2x higher intensities, and will thus evoked greater discomfort and pain. Thus, in L 161 “painful stimuli and” should be removed.

RESPONSE: We remove “painful stimuli and” from the manuscript.

Q: The sentence “To enable comparisons between sites, reported pain at L3 spinous process at 2 times the NWR threshold was matched between sites by adjustment of stimulation intensity” is not very clear to me. Because I believe it is not the NWR threshold that is matched (it is not recorded for most sites), but the stimulation intensity to elicit the reflexes was matched based on the perceived pain? I suggest a sentence similar to “To enable comparisons between sites, the stimulation intensity at other sites was adjusted to elicit the same degree of pain as stimulation at 2x NWR threshold at L3.”

RESPONSE: We agree with Reviewer 1 and modified the manuscript accordingly.

Q: In Table2 the authors report no significant main effect but still a p value of 0.09, indicating there is a quite a bit of variation in the perceived pain. Thus the sentence “It is noteworthy that the pain elicited by the electrical stimulus was not reported to differ between CLBP and CTL despite the use of larger intensity of stimulation in CLBP. ” should perhaps be soften up slightly to indicate that T2 indicate that the data are close to showing significant differences.

RESPONSE: In line with the Editor’s recommendation, we did not modify the manuscript.

*“In that regard, although I agree with most of the Reviewer's suggestions, I must disagree with the one regarding the interpretation of near-significant results(Table 2, p=0,09). While It might be tempting to discuss such results as 'significant' trends, we should avoid such interpretation and just report that no differences existed when the analysis failed to reach significance.”*

Q: Response 3: The use of ‘objective #2’ is not very clear, if this is has not been used previously in the MS. In the introduction the authors call them aims, consider using the same wording.

RESPONSE: We change the “objective” for “aim”.

Q: Regarding response 4: What was the definition of “response was evoked in the LM EMG”. What it based on an amplitude, peak2peak amplitude above X, or z-score or similar?

RESPONSE: Visual identification of the response was used. Visual identification of the reflex response was the gold standard at which the objective criteria from the study of Rhudy and France (2007) were compared to determine the absence/presence of NWR. We did not use objective criteria for reasons named in the previous review: (e.g. presence of artefact in the unfiltered EMG signals, low signal-to-noise ratio for EMG of low back muscles, differences in muscle mass, electrode positioning, and complexity of back muscle anatomy). We added a sentence in the Methods to clarify.

Methods:

p. 6; Ln 147-153: “To determine the NWR threshold, the current intensity began at 2 mA with the probe placed at L3. This was increased by 2-mA increments until a response was evoked in the LM EMG recording within the 40-200-ms window post-stimulation [35]. In the case of participants with high threshold, 5-mA increment was used. Although this was generally characterized by excitation, inhibition was sometimes observed as the earliest response when the participant was sitting. Motor response was identified visually when EMG clearly increases/decreases in comparisons to the background EMG signal (i.e. prior to stimulation). Visual identification from an expert is the gold standard to identify evoked responses [36].

Q: Response 5: please clarify that the number excluded (n) is referring to entire subjects and not just single reflexes.

RESPONSE: We refer to participants. However, this section has been removed since we removed the early window analysis from the manuscript.

Q: p. 6; Ln 148-156: Please be more specific as to what is ‘small increments’. Normally this is either a fixed step size or an adaptive step which changes with each deflection point (reflex present / not present).

RESPONSE: Usually, 2 mA was used as small increments. However, in the case of participants with high NWR threshold, 5 mA was used. This was added in the Mehods.

Methods

p.6; ln 148-150: “This was increased by 2-mA increments until a response was evoked in the LM EMG recording within the 40-200-ms window post-stimulation [35]. In the case of participants with high threshold, 5-mA increment was used.”

Q: The sentence in the discussion “Third, we were unable to measure the early response amplitude from some participants due to contamination of the early response by electrical.” Should be corrected, I believe “stimulation artefacts “ might be missing?

RESPONSE: We added “stimulation artifact” accordingly.

Q: p. 21, Ln 449-455: “It is important to note we did not assess Rib NWR threshold individually.” I suggest to underline to include a sentence similar to “NWR threshold was not individually assessed individually at each stimulation site.”

RESPONSE: We modified as suggested by Reviewer 1.

Q: Figure 2. thank you for including this figure. I think it is crucial to see this. Please indicate onset of stimulus. Please clarify what the grey traces represent. I suggest using an x-axis rather than a scaling bar, timing is a critical parameter to understand in relation to the NWR, especially when doing an early and late component analysis. Could you indicate the ‘response windows’? I believe this was 40-80 ms for the early component? And in relation to this, I notice that you have significant ringing after the stimulation artefact, it is important to ensure that these did not cover any of the analyzed reflex windows. Because based on fig 2 the ringing is still present beoynd 50 ms? This is hugely problematic and will effect the analysis of the early components. If ringing is present to this degree, I fear the analysis of the early component is invalid.

RESPONSE: Reviewer 1 is right if the artifact is present in the early window, it invalids the amplitude of the response. Fig 2 presents EMG signals from the muscles closest to the stimulation site (LM from S1), then, the impact of the artifact is larger for these muscles. Although we took care to remove all signals that were impacted by the stimulus artifact, we decided to remove the analysis of the amplitude of the early component, considering that some analyses were computed with very few participants, especially for participants with CLBP for which we use high current intensity. However, we think this is reasonable to keep our analysis of the occurrence of reflex since it was possible to identify them despite the stimulation artifact. The manuscript and figures were modified accordingly.

Q: Fig 2 legend: I do not understand the sentence “Note the large artifact that may influence EMG signal but the possibility to identify reflex occurrences.”

RESPONSE: The Fig 2 legend was modified. The meaning of the ble and grey traces were added.

Fig 2 legend:

***Fig 2*** *Examples of* *EMG* *activation* of *LM after S1 stimulation (upper panels) and EO after rib stimulation (lower panels) in controls (left panels) and participants with CLBP (right panels). The blue traces represent the EMG trace of all stimulation trials averaged. The grey traces represent single trials. The grey boxes represent the late windows (80-200 ms). Large artifact influences EMG signal although it did not impede the identification of early reflex occurrences. LM: Lumbar multifidus; OE: obliquus externus abdominis; S1: first spinous process of the sacrum.*

**Response to Reviewer #2:**

Thanks for addressing my comments. One small comment - in Table 1 there is a superscript 2 in the column with P values in the Gender row, but this is not mentioned in the legend, whereas the superscript 1 is (1Fisher Exact test used.)

RESPONSE: This was changed.
